# Plasma quercetin metabolites are affected by intestinal microbiota of human microbiota-associated mice fed with a quercetin-containing diet

**DOI:** 10.3164/jcbn.19-45

**Published:** 2019-10-10

**Authors:** Motoi Tamura, Hiroyuki Nakagawa, Sachiko Hori, Tadahiro Suzuki, Kazuhiro Hirayama

**Affiliations:** 1 Food Research Institute, National Agriculture and Food Research Organization, 2-1-12 Kannondai, Tsukuba, Ibaraki 305-8642, Japan; 2 Laboratory of Veterinary Public Health, Graduate School of Agricultural and Life Sciences, The University of Tokyo, 1-1-1 Yayoi, Bunkyo-ku, Tokyo 113-8657, Japan

**Keywords:** quercetin, intestinal microbiota, human microbiota-associated mice (HMA), LC-MS/MS

## Abstract

Protective effect of quercetin on high-fat diet-induced non-alcoholic fatty liver disease in mice has been reported. Recent research has revealed that several intestinal bacteria metabolize quercetin. We hypothesize that the difference in composition of intestinal microbiota affects quercetin absorption from the intestine. Germ-free BALB/cA female mice (18 weeks old) were randomly divided into four groups and orally administered with fecal suspension from four human individuals (HF1, HF2, HF3, HF4) to produce the human microbiota-associated mice. All mice were fed the 0.05% quercetin-containing pelleted diet for four weeks. Significant differences were observed in plasma total cholesterol and cecal microbiota among the four groups. Plasma quercetin concentration was significantly higher in the HF3 group than in the HF1 group. The plasma isorhamnetin/quercetin ratio showed significant negative correlation with visceral fat levels (*r* = −0.544, *p* = 0.013). Positive correlation was observed between the Log_10_ *Enterobacteriaceae* count and the plasma quercetin metabolites. Principal component analysis showed that all groups were distributed in different regions by using the correlation diagram with the second and third principal component. This study indicates that intestinal microbiota of human microbiota-associated mice inoculated with different fecal suspensions react to dietary quercetin in different ways.

## Introduction

Much attention has been focused on the health promoting effects of quercetin. Quercetin is one of the major flavonoids found in plants such as onion, tea, and asparagus. Several beneficial effects of quercetin have been reported. The antioxidant capacity of quercetin metabolites under physiological conditions was reported.^(1)^ It has been reported that vitamins C and E protect against alcohol-mediated toxic effects during liver regeneration in rat.^(2)^ Quercetin also prevents alcohol-induced liver injury in mice.^(3)^ Protective effects of quercetin on high-fat diet-induced non-alcoholic fatty liver disease (NAFLD) in mice^(4)^ and rat^(5)^ have been reported. Intake of quercetin alleviates hepatic fat accumulation and decreases accumulation of hepatic and circulating lipids in mice.^(6)^ It has been reported that quercetin reduces blood pressure in hypertensive subjects.^(7)^ Cancer prevention by quercetin has also been reported.^(8)^ Quercetin’s cancer suppression effects were partly through inhibition of inflammatory mediators.^(9)^ Anti-allergic effects of quercetin were characterized by its stimulation of the immune system.^(10)^ Thus, quercetin acts as important functional food.

On the other hand, quercetin affects the function of gut bacteria. It has been reported that *Bifidobacterium adolescentis* co-cultured with quercetin or other flavonols (galangin and fisetin) highly suppressed nitric oxide (NO) production in lipopolysaccharide-stimulated RAW264 cells.^(11)^ Prebiotic dietary fiber helps improve constipation and gut dysbiosis symptoms and behavioral irritability in children with autism spectrum disorder.^(12)^ Flavonols like quercetin are believed to have a prebiotic-like effect on the anti-inflammatory activity of *Bifidobacterium adolescentis*.^(11)^ Dietary quercetin is expected to improve the intestinal microbiota.

Quercetin is degraded by intestinal bacteria. Quercetin was degraded by *Eubacterium ramulus*^(13)^ and *Clostridium orbiscindens*.^(14)^ Quercetin can also be degraded by *Clostridium perfringens* and *Bacteroides fragilis*.^(15)^ It has been indicated that the [C^14^]-quercetin administered to rats were considerably degraded in the digestive tract.^(16)^

Recent research has revealed that many kinds of intestinal bacteria metabolize quercetin.^(15)^ As intestinal microbiota seems to affect the host physiological function and metabolism of quercetin, elucidation of the effects of intestinal microbiota on quercetin metabolism is one of the important research fields. However, there are few reports showing effects of microbiota on bioavailability of quercetin. We hypothesized that intestinal microbiota affects absorption of quercetin. To confirm this hypothesis, we produced the human flora-associated mice from four different human feces, and we investigated the plasma quercetin and lipid profiles in these four different human microbiota-associated mouse groups fed with dietary quercetin.

## Materials and Methods

### Sampling of human feces

Feces from four healthy adult Japanese females who did not have any gastrointestinal disease (21–54 years of age) (HF1, HF2, HF3, HF4) were individually collected on paper sheets and immediately transferred into sterilized containers (Sarstedt K.K., Tokyo, Japan) that were placed in an AnaeroPouch with a CO_2_ generator (Mitsubishi Gas Chemical Company), Tokyo, Japan and these feces were stored in the freezer at −80°C. This study was performed under the guidelines of the Helsinki Declaration (1964). The Human Investigations Review Board of the Food Research Institute approved the study protocol and informed consent was obtained from the subjects.

### Animals and diets

Germ-free BALB/cA female mice were bred and maintained in the Laboratory of Veterinary Public Health, University of Tokyo. Before feeding the mice the experimental diets, animals were fed the AIN-93M pelleted diet purchased from Oriental Yeast Co., Ltd. (Tokyo, Japan). At the start of the experiment, 18-weeks-old germ-free mice were randomly divided into four groups of five animals each. Fecal samples (HF1, HF2, HF3, HF4) were thawed and weighed into sterile glass homogenizers to prepare 100-fold dilutions with the anaerobic medium in an anaerobic chamber. The mice were orally inoculated with these dilutions into the stomach with a metal feeding needle in the flexible vinyl isolator. All mice were fed the AIN-93M pelleted diet for three weeks, followed by a 0.05% quercetin-containing pelleted diet for four weeks. The composition of the quercetin-containing diet was 14% casein, 0.18% l-cystine, 38.8179% corn starch, 16.7% α-corn starch, 10% sucrose, 3% soy bean oil, 7.5% lard, 5% cellulose powder, 3.5% AIN-76 mineral mix, 1% AIN-93 vitamin mix, 0.25% choline bitartrate, 0.0021% *tert*-butylhydroquinone, and 0.05% quercetin. Both the AIN-93M and 0.05% quercetin-containing diets were sterilized by γ-irradiation at 50 kGy and the final body weight was measured. Three days before the end of the experimental diet-feeding, feces were collected individually from all mice and stored at −80°C until analysis of bile acids. After the experimental diet-feeding period, the mice were anesthetized and sacrificed by exsanguination and blood samples were taken from the heart and placed in heparinized tubes. The plasma was separated from whole blood by centrifugation and stored at −80°C until analysis of plasma quercetin metabolites and plasma lipids. The liver, visceral fat, and cecal contents were collected and weighed. Liver samples were stored at −80°C until liver lipids analysis. All procedures involving mice in this study were approved by the Animal Care Committee of the University of Tokyo, in accordance with guidelines from the “Guidelines for Animal Care and Experimentation” of the University of Tokyo.

### Measurement of plasma cholesterol, triglyceride, non-esterified fatty acid (NEFA)

The following tests were performed with kits purchased from FUJIFILM Wako Pure Chemical Corporation (Osaka, Japan). Total plasma cholesterol concentrations were measured using a cholesterol E-test kit based on cholesterol oxidase. Plasma triglyceride concentrations were determined using a triglyceride E-test kit based on the glycerol-3-phosphate oxidase method. Plasma non-esterified fatty acid (NEFA) concentrations were measured using a NEFA C-test kit involving the acyl-coenzyme A (CoA) synthase, acyl-CoA oxidase, and peroxidase method.

### Measurements of amounts of fecal bile acid

Feces were dried with a freeze dryer (FD-1000; Tokyo Rikakikai Co., Ltd., Tokyo, Japan) for 24 h. The trap cooling temperature was −45°C. After drying, weights of freeze-dried feces were measured and the samples were ground in a mortar. Fecal amounts of bile acid were measured by the method described by Kanamoto *et al.*^(17)^ A total of 10 mg of feces were suspended in a glass test tube with 2.5 ml of 99.5% ethanol, vortexed for 30 s, incubated for 1 h at 65°C, and centrifuged at 3,000 rpm for 10 min at 4°C. The supernatants were transferred to a glass test tube. The same volume (2.5 ml) as that used in the first extraction of 99.5% ethanol was added to the sediment and the procedure was repeated. The supernatants from both extractions were pooled in the same glass test tube and dried at 65°C gassing with N_2_ gas. After drying, 0.5 ml of 90% ethanol was added to the residue and vortexed for 30 s. Total bile acid concentrations were measured with a total bile acid test (FUJIFILM Wako Pure Chemical Corporation, Osaka, Japan) according to the manufacturer’s instructions.

### Measurements of lipids, triglyceride and cholesterol in the liver

Liver lipids were extracted using the Bligh and Dyer method.^(18)^ The extracted liver lipid was dissolved in 2-propanol containing 10% Triton X-100. Liver cholesterol and triglyceride concentrations were measured using the same methods as plasma cholesterol and triglyceride.

### Analysis of plasma quercetin metabolites

Plasma quercetin and isorhamnetin were analyzed according to the method described by Kashino *et al.*^(19)^ with some modifications. A total of 50 µl of plasma was added to 50 µl of β-glucuronidase H-5 solution (35 mg/ml; Sigma, MO) in 0.1 M sodium acetate buffer (pH 5.0). The mixture was incubated at 37°C in a water bath for 40 min, followed by treatment with 200 µl of ethyl acetate, vortexing for 30 s, and centrifugation at 11,000 × *g* for 10 min at 4°C. The supernatants were transferred to the glass test tube. The same volume of ethyl acetate as the first extraction was added to the sediment, and the procedure was repeated. The supernatants from both extractions were pooled into the same glass test tube and dried completely by N_2_ gassing at room temperature. The sample was then dissolved in 200 µl of 80% methanol and centrifuged at 11,000 × *g* for 10 min. The supernatants were subjected to LC-MS/MS analysis. Detection and quantification was performed with a 4000 QTRAP LC-MS/MS system (AB Sciex, Foster City, CA) equipped with an atmospheric pressure chemical ionization (APCI) source (heated nebulizer) and 1100 Series HPLC system (Agilent, Waldbronn, Germany). Chromatographic separation was performed at 40°C on a ZORBAX Eclipse Plus C18 column, 150 × 2.1 mm i.d., 3.5 µm particle size (Agilent). Eluents were composed of water/acetic acid (99.9:0.1, v/v) containing 0.5 mM ammonium acetate (eluent A), and acetonitrile/acetic acid (99.9:0.1, v/v) (eluent B), respectively. Both solvents were prepared with chemicals of LC/MS grade (water, acetonitrile) and HPLC grade (acetic acid). Elution was conducted at a flow rate of 0.2 ml/min with a linear gradient of acetonitrile. After maintaining B at 5% for 2 min, it was increased linearly to 95% over 15 min, followed by a hold time of 5 min at 95% B. Thereafter, the proportion of B was decreased to 5% over 1 min, and kept at 5% for 10 min prior to the next sample injection. APCI-MS/MS was performed in multiple reaction monitoring (MRM) mode at positive polarity with the following settings: source temperature 400°C, curtain gas 10 psi, ion source gas 1 (sheath gas) 60 psi, ion source gas 2 (drying gas) 10 psi, needle current 3 µA, and dwell time 20 ms. The optimization of the analyte-dependent MS/MS parameters was performed by direct infusion of standards. Each analyte dissolved in MeOH (1–10 mg/L) was supplied at the rate of 10 µl/min with a syringe pump, mixed via a “mixing T” with the carrier solvent adjusted to 50% B, and introduced into the mass spectrometer at a flow rate of 0.2 ml/min. MS and MS/MS parameters (such as the selection of the most abundant MRM transitions, declustering potentials, collision energies, and cell exit potentials) were optimized for all analytes in the positive APCI mode.

### DNA extraction from cecal contents

Procedures of DNA extraction from cecal contents were conducted according to Matsuki’s method.^(20)^ Cecal samples (20 mg) were washed two times by resuspending them in 0.2 ml of PBS and centrifuging each preparation at 14,000 × *g* to remove possible PCR inhibitors. Following the second centrifugation, the cecal pellets were resuspended in a solution consisting of 0.2 ml of PBS, 250 µl of extraction buffer (200 mM Tris-HCl, 80 mM EDTA. pH 9.0), and 50 µl of 10% sodium dodecyl sulfate. A total of 300 mg of glass beads (diameter 0.1 mm) and 500 µl of buffer-saturated phenol were added to the suspension, and the mixture was vortexed vigorously for 180 s using a MicroSmash (Tomy Seiko Co., Ltd, Tokyo, Japan) at 4,000 rpm. After centrifugation at 14,000 × *g* for 5 min, 400 µl of the supernatant was collected. Phenol–chloroform–isoamyl alcohol extraction was then performed, and 250 µl of the supernatant was subjected to isopropanol precipitation. Finally, the obtained DNA was dissolved in 1 ml of 10 mM Tris-EDTA buffer, pH 8.0. The DNA solution was adjusted to a final concentration of 10 µg/ml in the same buffer.

### Real-time PCR

qPCR was carried out on a Real-Time QPCR System Mx3000p (Agilent Technologies Ltd., Santa Clara, CA) to determine *Bifidobacterium*, *Clostridium leptum* subgroup, *Clostridium coccoides* group, *Bacteroides fragilis* group, *Atopobium* cluster, and *Enterobacteriaceae* cell counts by means of specific primers (Table 1). Next, 10 ng of DNA from cecal contents (1 µl) was added to 19 µl of the reaction mix [0.4 µl of each 10 µM primer, 8.2 µl of distilled water and 10 µl of 2× KAPA SYBER FAST qPCR Master Mix Universal (Kapa Biosystems Inc., Wilmington, MA)].

The amplification program for the *Atopobium* cluster consisted of one cycle of 95°C for 3 min and then 40 cycles of 95°C for 3 s, 62°C for 60 s. The cycling conditions for the *Bacteroides fragilis* group involved one cycle of 95°C for 3 min and then 40 cycles of 95°C for 3 s and 58°C for 60 s. The amplification program for *Bifidobacterium* consisted of one cycle of 95°C for 3 min and then 40 cycles of 95°C for 3 s, and 58°C for 60 s. The amplification program for *Clostridium leptum* subgroup consisted of one cycle of 95°C for 3 min and then 40 cycles of 95°C for 3 s, 60°C for 60 s. The amplification program for the *Clostridium coccoides* group consisted of one cycle of 95°C for 3 min and then 40 cycles of 95°C for 3 s and 60°C for 60 s. The thermal cycling conditions for *Enterobacteriaceae* included one cycle of 95°C for 3 min and then 40 cycles of 95°C for 5 s, 66°C for 20 s, and 72°C for 40 s. To check the specificity of PCR, a melting curve analysis was conducted after the amplification. The melting curves were obtained by heating at temperatures from 55 to 95°C with continuous fluorescence monitoring. DNA samples extracted from *Clostridium clostridioforme* JCM1291^T^, *Bacteroides thetaiotaomicron* Tok6-10, *Bifidobacterium longum* subsp. *longum* JCM1217^T^, *Eggerthella lenta* JCM9979^T^, *Escherichia coli* JCM 20135, and *Faecalibacterium prausnitzii* JCM 31915 served as real-time PCR standard for the group-specific g-Ccoc, g-Bfra, g-Bifido, c-Atopo, Eco, sg-Clept primers, respectively. A standard curve was generated with the RT-qPCR data and the corresponding standard cell count. For enumeration of standard bacteria, we used the Bacstain DAPI solution (Dojindo Laboratories., Kumamoto, Japan) based on 4',6-diamidino-2-phenylindole (DAPI) staining according to the manufacturer instructions and then trapped between a glass slide and a square coverslip. The cells were imaged with a fluorescence microscope (BZ-8000; KEYENCE CORPORATION, Osaka, Japan). Images were then produced by using the image analysis software ImageJ 1.52a (National Institute of Health, Bethesda, Maryland), and the total number of cells detected in each field was calculated.

### Statistics

Data are expressed as mean ± SE. All data were analyzed using Sigma Plot 11 (Systat Software, Inc., San Jose, CA) by the one-way analysis of variance. In the multiple comparison, the Tukey test was used. Statistical significance was assumed at a *p* value <0.05. For correlation analysis, we used the Spearman Rank Order Correlation. We performed principal component analysis of the data using the online website of Gunma University (http://aoki2.si.gunma-u.ac.jp/BlackBox/BlackBox.html) for statistical analysis.

## Results

No significant differences were observed among four groups in final body weight [g; HF1 (27.4 ± 1.5), HF2 (28.8 ± 0.9), HF3 (26.7 ± 0.5), HF4 (29 ± 1.2)], liver weight [g; HF1 (1.34 ± 0.08), HF2 (1.35 ± 0.04), HF3 (1.33 ± 0.06), HF4 (1.31 ± 0.06)], visceral fat [g; HF1 (1.50 ± 0.36), HF2 (2.00 ± 0.19), HF3 (1.32 ± 0.09), HF4 (1.90 ± 0.31)], food consumption [g; HF1 (3.6 ± 0.06), HF2 (3.6 ± 0.04), HF3 (3.7 ± 0.02), HF4 (3.6 ± 0.20)]. Cecal contents (g) were HF1 (0.16 ± 0.01), HF2 (0.19 ± 0.02), HF3 (0.28 ± 0.01), HF4 (0.19 ± 0.01). The cecal contents of HF3 group was significantly greater than the other three groups (*p*<0.05).

The concentrations of triglyceride, total cholesterol, and NEFA, and amounts of liver lipids, liver cholesterol, and liver triglyceride were shown in Table 2. Plasma total cholesterol levels were significantly lower in HF3 group (133.3 ± 5.4) than in the HF2 group (163.8 ± 6.1). There were no significant differences in plasma triglyceride and NEFA levels between the four groups. There were also no significant differences in liver lipid, liver cholesterol and liver triglyceride levels between the four groups.

**Fig. 1:**
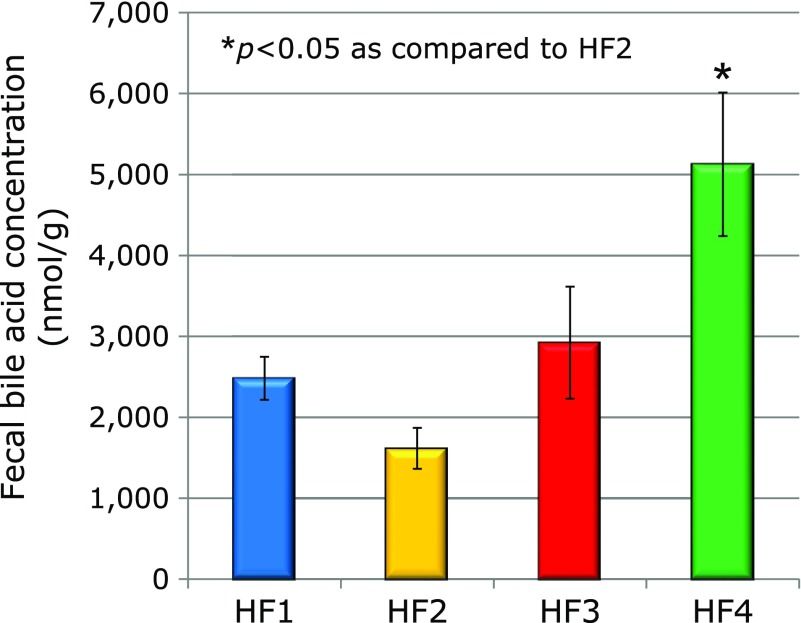
Fecal bile acid concentration (nmol/g) from the HMA mice in the HF1, HF2, HF3, and HF4 group. Values are means ± SE (*n* = 5). *Significantly different from the HF2 group (*p*<0.05).

Fecal bile acid concentrations are shown in Fig. 1. There was significant difference in the fecal bile acid concentration between four groups. Average fecal bile acid concentration (nmol/g feces) was greatest in HF4 group amongst the four groups. Fecal bile acid concentration was significantly greater in HF4 group (5,124.7 ± 887.4) than in the HF2 group (1,616.9 ± 253.9).

**Fig. 2:**
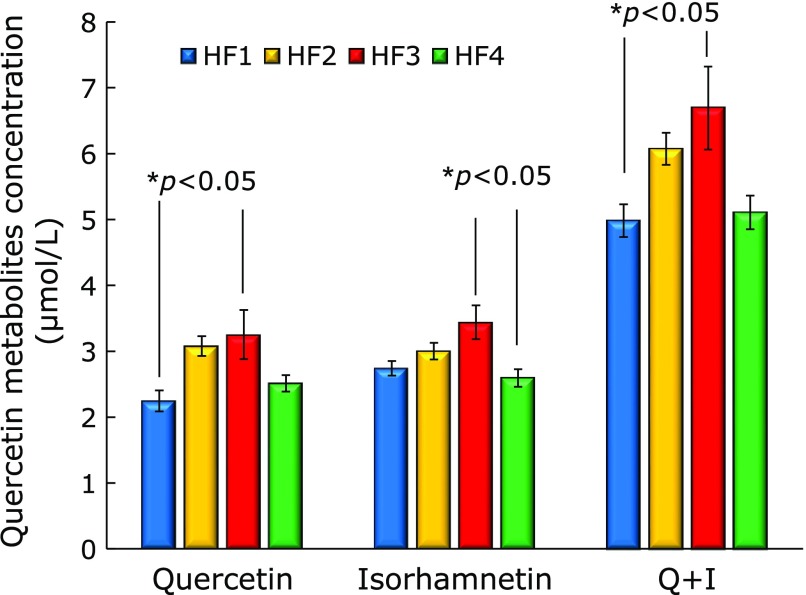
Plasma quercetin metabolite concentrations (µmol/L) from the HMA mice in the HF1, HF2, HF3, and HF4 group. Values are means ± SE (*n* = 5). The data were analyzed using All Pairwise Multiple Comparison Procedures (Tukey test) *Significantly different (*p*<0.05).

There were significant differences in the quercetin metabolites between four groups (Fig. 2). Plasma quercetin concentration (µmol/L) was significantly greater in the HF3 group (3.25 ± 0.37) than in the HF1 group (2.24 ± 0.16). Isorhamnetin (3'-methylquercetin) is one of quercetin metabolites found in the liver and plasma isorhamnetin concentration was significantly greater in the HF3 (3.44 ± 0.26) group than in HF4 group (2.6 ± 0.13). Plasma quercetin plus isorhamnetin concentration was significantly greater in the HF3 (6.69 ± 0.63) group than in the HF1 group (4.98 ± 0.25).

**Fig. 3:**
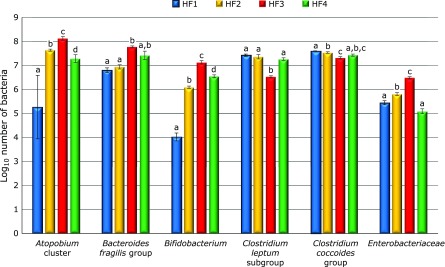
The cell number of cecal *Atopobium* cluster, *Bacteroides fragilis* group, *Bifidobacterium*, *Clostridium coccoides* group, *Clostridium leptum* subgroup, and *Enterobacteriaceae* per µg of DNA from cecal contents. Significant difference between alphabets with different superscript (*p*<0.05).

The cell number of cecal *Atopobium* cluster, *Bacteroides fragilis* group, *Bifidobacterium*, *Clostridium coccoides* group *Clostridium leptum* subgroup, and *Enterobacteriaceae* per µg of DNA from cecal contents are shown in Fig. 3. The intestinal microbiota was different among the 4 groups. Cecal *Atopobium* cluster counts were significantly greater in the HF3 group than in the other three groups. Cecal *Bacteroides fragilis* group counts were significantly greater in the HF3 group than in the HF1 and HF2 groups. Cecal *Bifidobacterium* counts were significantly greater in the HF3 group than in the other three groups. Cecal *Clostridium leptum* subgroup counts were significantly lower in the HF3 group than in the other three groups. Cecal *Clostridium coccoides* group counts were significantly greater in the HF1 group than in the HF2 and HF3 groups. Cecal *Enterobacteriaceae* counts were significantly greater in the HF3 group than in the other three groups.

**Fig. 4:**
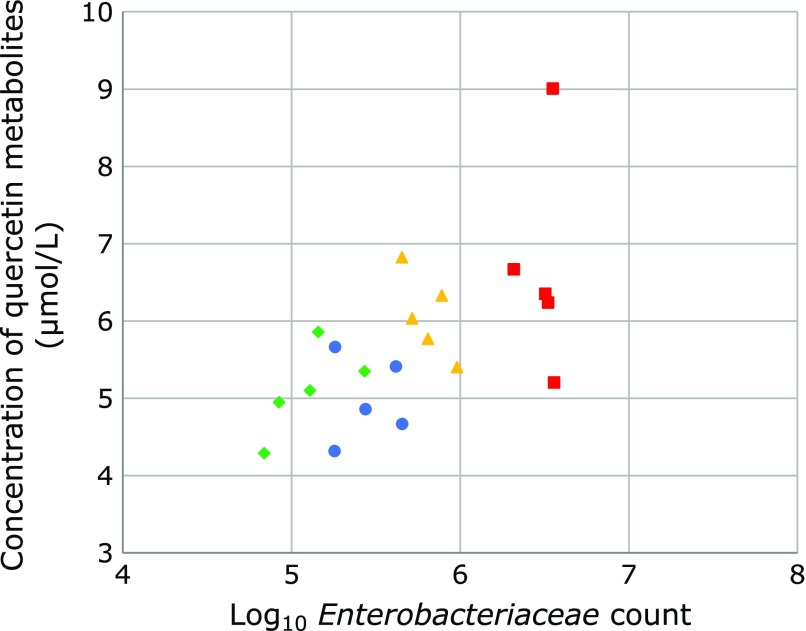
Correlation between the Log_10_ *Enterobacteriaceae* count and the plasma quercetin metabolites (quercetin and isorhamnetin). Positive correlation (*r* = 0.618, *p* = 0.007) was observed between the Log_10_ *Enterobacteriaceae* count and the plasma quercetin metabolites (quercetin and isorhamnetin) (circle, HF1 group; triangle, HF2 group; square, HF3 group; rhombus, HF4 group).

Positive correlation (*r* = 0.618, *p* = 0.007) was observed between the Log_10_ *Enterobacteriaceae* count and the plasma quercetin metabolites (quercetin and isorhamnetin) (Fig. 4). Weak positive correlation (*r *= 0.38, *p* = 0.096) was observed between the Log_10_ *Bifidobacterium* count and the plasma quercetin metabolites (quercetin and isorhamnetin).

**Fig. 5:**
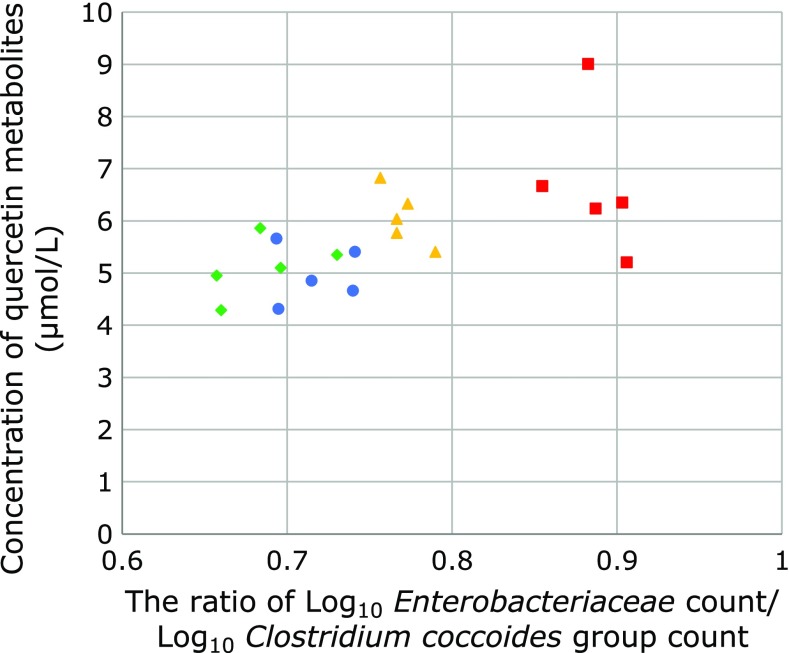
Correlation between the ratio of Log_10_ *Enterobacteriaceae* count/Log_10_ *Clostridium coccoides* group count and the plasma quercetin metabolites. Positive correlation (*r* = 0.606, *p* = 0.008) was observed between the ratio of Log_10_ *Enterobacteriaceae* count/Log_10_ *Clostridium coccoides* group count and the plasma quercetin metabolites. (circle, HF1 group; triangle, HF2 group; square, HF3 group; rhombus, HF4 group).

**Fig. 6:**
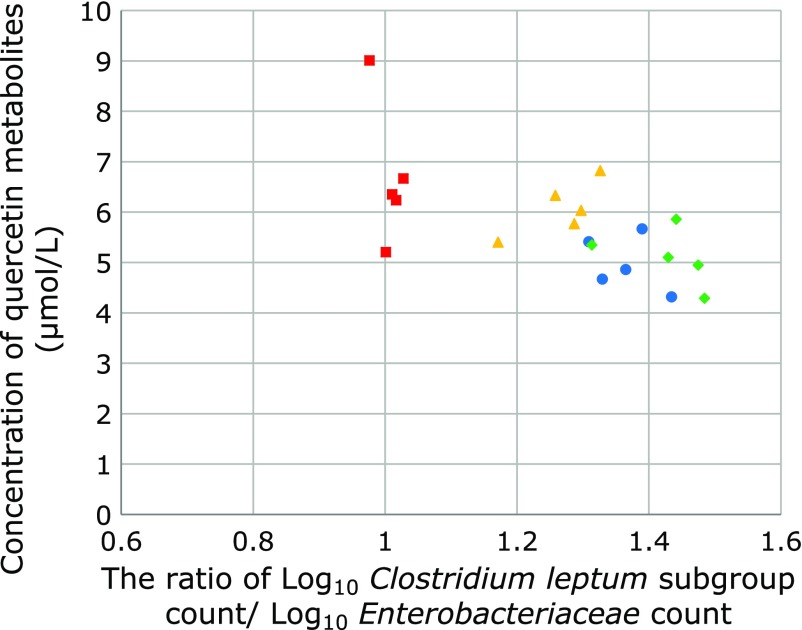
Correlation between the ratio of Log_10_ *Clostridium leptum* subgroup count/Log_10_ *Enterobacteriaceae* count and the plasma quercetin metabolites. Negative correlation (*r* = –0.642, *p* = 0.005) was observed between the ratio of Log_10_ *Clostridium leptum* subgroup count/Log_10_ *Enterobacteriaceae* count and the plasma quercetin metabolites. (circle, HF1 group; triangle, HF2 group; square, HF3 group; rhombus, HF4 group).

Positive correlation (*r* = 0.606, *p* = 0.008) was observed between the ratio of Log_10_ *Enterobacteriaceae* count/Log_10_ *Clostridium coccoides* group count and the plasma quercetin metabolites (Fig. 5). On the other hand, negative correlation (*r* = −0.642, *p* = 0.005) was observed between the ratio of Log_10_ *Clostridium leptum* subgroup count/Log_10_ *Enterobacteriaceae* count and the plasma quercetin metabolites (Fig. 6).

**Fig. 7:**
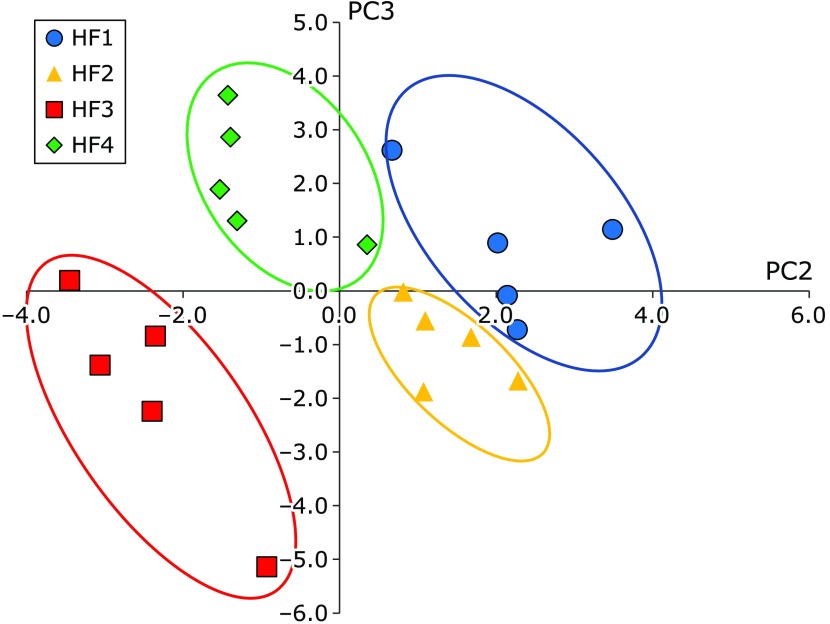
Distribution on the third principal component (y axis) and the second principal component (x axis) of the principal component score using the correlation diagram. All four groups are clearly divided into four groups. (circle, HF1 group; triangle, HF2 group; square, HF3 group; rhombus, HF4 group).

Principal component analysis was performed by using the data of cecal number of bacteria, plasma total cholesterol, triglyceride, NEFA, liver lipids, liver cholesterol, liver triglyceride, fecal bile acids, plasma quercetin, plasma isorhamnetin, and plasma quercetin plus isorhamnetin. Contribution rate was 20.65 for principal component 1, 20.20 for principal component 2, 19.61 for principal component 3, and 12.28 for principal component 4. Distribution on the third principal component (y axis) and the second principal component (x axis) of the principal component score of HF1 group, HF2 group, HF3 group and HF4 group are clearly divided into four groups on the correlation diagram (Fig. 7).

## Discussion

In our experiment, although the quercetin content of the experimental diet was the same for all groups, plasma quercetin metabolites in HF3 was significantly more than in HF1 group.

It has been reported that the microbiota degrade quercetin to produce phenolic degradation products,^(24)^ and many intestinal bacteria have different quercetin degrading ability.^(25)^ Thus, quercetin metabolism by intestinal microbiota plays an important role in absorption of quercetin. Because inter-individual variation in quercetin metabolism by intestinal microbiota has been suggested,^(26)^ plasma quercetin concentration of HMA mice might be affected by the degree of quercetin metabolism by inoculated microbiota.

Isorhamnetin has been proposed to be a quercetin metabolite in the liver.^(16)^ The ratio of plasma isorhamnetin/quercetin showed significant negative correlation with amounts of visceral fat (*r* = −0.544) (Table 3) although plasma isorhamnetin or quercetin concentration did not (Table 3). It has been reported that accumulation of visceral fat correlates with the metabolic syndrome occurrence.^(27)^ The present study suggests that relative concentration of isorhamnetin against quercetin might relate to the occurrence of metabolic syndrome. Isorhamnetin is suggested to be a quercetin metabolite formed by a liver enzyme.^(16)^ Quercetin circulating in plasma would be metabolized to a greater extent to isorhamnetin in the liver when quercetin circulates longer in blood. So, higher ratio of plasma isorhamnetin/quercetin might mean longer blood circulation time. On the other hand, HMA mice with a high ratio of plasma isorhamnetin/quercetin might have higher quercetin methylation enzyme activity in liver than those with a low ratio.

NAFLD is considered as a hepatic complication of metabolic syndrome.^(28)^ Higher ratio of plasma isorhamnetin/quercetin might contribute to the suppression of the development of NAFLD. It has been reported that quercetin alleviates metabolic syndrome.^(6)^ Our results suggest that the ratio of plasma isorhamnetin/quercetin, but not merely the concentration of quercetin or isorhamnetin in plasma, is more relevant to anti-metabolic syndrome effects of dietary quercetin. Further studies on this mechanism would be necessary in future.

Fecal bile acid concentrations were significantly greater in the HF4 group than those in the HF2 groups. Bile acids are reabsorbed from the ileum as conjugates.^(29)^ Some kinds of intestinal bacteria deconjugate the bile acid conjugate and affect the resorption of bile acid.^(30,31)^ Thus, the composition of intestinal microbiota may affect fecal excretion of bile acid.

Through principal component analysis, we found that all groups were distributed in independent regions by using the correlation diagram with the second and third principal component. It is inferred that each of these four groups have different characteristics and no intersection of regions. Principal component 3 is defined as a plasma quercetin metabolite lowering factor, and principal component 2 is defined as a microbiota related factor. The HF1 and HF4 groups have similarity in the plot distribution in the correlation diagram with the third principal component. In fact, these two groups share similar tendency in plasma quercetin and isorhamnetin level.

There were significant differences in the quercetin metabolites between four groups. Just as there are individual differences in intestinal microbiota, there might be individual differences in quercetin metabolism. As the result of principal component analysis, we suppose that different type of microbiota differently affects the plasma quercetin metabolites. Positive correlation was observed between the Log_10_ *Enterobacteriaceae* count and the plasma quercetin metabolites (quercetin and isorhamnetin). It has been reported that *Enterobacteriaceae* were positively correlated with quercetin concentration in the human fecal incubation solution with quercetin.^(26)^ Positive correlation was observed between the ratio of Log_10_ *Enterobacteriaceae* count/Log_10_ *Clostridium coccoides* group count and the plasma quercetin metabolites. On the other hand, negative correlation was observed between the ratio of Log_10_ *Clostridium leptum* subgroup count/Log_10_ *Enterobacteriaceae* count and the plasma quercetin metabolites. It has been reported that the fate of quercetin in the lower gut depends on the composition of microbiota that metabolize this compound.^(26)^ The composition of the microbiota may be related to the bioavailability of quercetin metabolites. Intestinal microbiota affects the quercetin degradation.^(13–15)^ Quercetin undergoes glucuronidation in the intestinal cells^(32)^ and direct biliary excretion of enterically or extrahepatically derived flavonoid glucuronides has been reported.^(33)^ Intestinal microbiota has glucuronidase activity and deconjugation of quercetin-glucuronide occurs by these bacterial glucuronidase.^(34)^ Thus, intestinal bacteria would affect the quercetin absorption in the lower gut.

Quercetin ameliorates high fat diet-induced NAFLD in male Sprague-Dawley rats.^(5)^ Protective effects of quercetin on high-fat diet-induced NAFLD in mice are reported.^(4)^ On the other hand, development of NAFLD is affected by the intestinal microbiota.^(35)^ Modulation of intestinal microbiota leads to a new therapeutic approach for NAFLD.^(36)^ In our experiment, plasma quercetin concentration in HF3 group was the highest in all groups. Average visceral fat weight was the lowest in HF3 group among all groups. The intestinal microbiota was significantly different among the 4 groups. The intestinal microbiota of HF3 group may be the most effective in preventing NAFLD. Prebiotic dietary fiber helps improve constipation and gut dysbiosis symptoms and behavioral irritability in children with autism spectrum disorder.^(12)^ Quercetin supposed to have a prebiotic-like effect.^(11)^ The present study has indicated that intestinal microbiota of human microbiota-associated mice inoculated with fecal suspensions from different donors react to dietary quercetin in different ways. Thus, it is speculated that intestinal microbiota strongly affects the bioavailability of quercetin. Intestinal microbiota that improves the bioavailability of quercetin may be effective in preventing NAFLD.

## Author Contribitions

MT and KH designed research. KH, MT, HN, SH, TS conducted research and analyzed data. MT wrote paper. All authors read and approved the final manuscript.

## Figures and Tables

**Table 1:** 16S rRNA gene-targeted group-specific primers

Target organism	Primers	Sequences (5' to 3')	Size (bp)	References
*Atopobium* cluster	c-Atopo-F	GGGTTGAGAGACCGACC	190	(21)
	c-Atopo-R	CGGRGCTTCTTCTGCAGG		
*Bacteroides fragilis* group	g-Bfra-F	ATAGCCTTTCGAAAGRAAGAT	495	(21)
	g-Bfra-R	CCAGTATCAACTGCAATTTTA		
*Bifidobacterium*	g-Bifid-F	CTCCTGGAAACGGGTGG	549–563	(22)
	g-Bifid-R	GGTGTTCTTCCCGATATCTACA		
*Clostridium leptum subgroup*	sg-Clept-F	GCACAAGCAGTGGAGT	239	(21)
	sg-Clept-R3	CTTCCTCCGTTTTGTCAA		
*Clostridium coccoides* group	g-Ccoc-F	AAATGACGGTACCTGACTAA	438–441	(22)
	g-Ccoc-R	CTTTGAGTTTCATTCTTGCGAA		
*Enterobacteriaceae*	Eco1457F	CATTGACGTTACCCGCAGAAGAAGC	195	(23)
	Eco1652R	CTCTACGAGACTCAAGCTTGC		

**Table 2:** The concentrations of plasma cholesterol, triglyceride, NEFA, amounts of liver lipids, liver cholesterol, and liver triglyceride

	HF1 group	HF2 group	HF3 group	HF4 group
Plasma cholesterol (mg/dl)	152.0 ± 4.8^a,b^	163.8 ± 6.1^a^	133.3 ± 5.4^b^	143.1 ± 7.7^a,b^
Plasma triglyceride (mg/dl)	170.8 ± 10.0	176.1 ± 10.7	170.8 ± 4.3	212.2 ± 51.9
Plasma NEFA (mEq/L)	2.5 ± 0.1	2.2 ± 0.06	2.5 ± 0.1	2.7 ± 0.4
Liver lipids (g)	0.11 ± 0.01	0.11 ± 0.01	0.10 ± 0.01	0.12 ± 0.01
Liver triglyceride (mg)	53.8 ± 7.9	62.7 ± 0.8	51.2 ± 7.2	66.3 ± 10.0
Liver cholesterol (mg)	7.0 ± 2.9	6.1 ± 1.0	5.6 ± 0.9	6.0 ± 0.5

Significant difference between alphabets with different superscript (*p*<0.05).

**Table 3:** Correlation between plasma quercetin, isorhamnetin, the ratio of plasma isorhamnetin/quercetin with visceral fat

Correlation with Visceral fat	Quercetin vs Visceral fat	Isorhamnetin vs Visceral fat	Isorhamnetin/Quercetin vs Visceral fat
Correlation coefficient	0.147	–0.108	–0.544*
*p* value	0.529	0.644	0.013

*Significantly different (*p*<0.05).
